# Evaluation of the Effect of Tumor Position on Standardized Uptake Value Using Time-of-Flight Reconstruction and Point Spread Function

**DOI:** 10.7508/aojnmb.2016.04.007

**Published:** 2016

**Authors:** Yasuharu Wakabayashi, Kenichi Kashikura, Yasuyuki Takahashi, Hitoshi Yabe, Akihiro Ichikawa, Souichi Yamamoto, Ayumi Ishii, Kunio Doi

**Affiliations:** 1 Division of Radiological Technology, Saitama Prefectural Cancer Center, Saitama, Japan; 2 Graduate School of Radiological Technology, Gunma Prefectural College of Health Sciences, Gunma, Japan; 3 Division of Health Sciences, Graduate School of Medical Sciences, Kanazawa University, Kanazawa, Japan; 4 Division of Molecular Imaging, Saitama Prefectural Cancer Center, Saitama, Japan; 5 Department of Radiology, University of Chicago, Chicago, Illinois, USA

**Keywords:** ^18^F-FDG, Point spread function, Standardized Uptake Value, Time-of-flight, Tumor size

## Abstract

**Objective(s):**

The present study was conducted to examine whether the standardized uptake value (SUV) may be affected by the spatial position of a lesion in the radial direction on positron emission tomography (PET) images, obtained via two methods based on time-of-flight (TOF) reconstruction and point spread function (PSF).

**Methods:**

A cylinder phantom with the sphere (30 mm diameter), located in the center was used in this study. Fluorine-18 fluorodeoxyglucose (^18^F-FDG) concentrations of 5.3 kBq/ml and 21.2 kBq/ml were used for the background in the cylinder phantom and the central sphere respectively. By the use of TOF and PSF, SUV_*max*_ and SUV_*mean*_ were determined while moving the phantom in a horizontal direction (X direction) from the center of field of view (FOV: 0 mm) at 50, 100, 150 and 200 mm positions, respectively. Furthermore, we examined 41 patients (23 male, 18 female, mean age: 68±11.2 years) with lymph node tumors, who had undergone ^18^F-FDG PET examinations. The distance of each lymph node from FOV center was measured, based on the clinical images.

**Results:**

As the distance of a lesion from the FOV center exceeded 100 mm, the value of SUV_*max*_, which was obtained with the cylinder phantom, was overestimated, while SUV_*mean*_ by TOF and/or PSF was underestimated. Based on the clinical examinations, the average volume of interest was 8.5 cm3. Concomitant use of PSF increased SUV_*max*_ and SUV_*mean*_ by 27.9% and 2.8%, respectively. However, size of VOI and distance from the FOV center did not affect SUV_*max*_ or SUV_*mean*_ in clinical examinations.

**Conclusion:**

The reliability of SUV quantification by TOF and/or PSF decreased, when the tumor was located at a 100 mm distance (or farther) from the center of FOV. In clinical examinations, if the lymph node was located within 100 mm distance from the center of FOV, SUV remained stable within a constantly increasing range by use of both TOF and PSF. We conclude that, use of both TOF and PSF may be helpful.

## Introduction

The spatial resolution of positron emission tomography (PET) images in the early stages of its clinical application was greater than 10 mm, which was inferior to other imaging modalities (1, 2). However, in recent years, various techniques have been proposed to improve the resolution of PET images (3). In particular, development of new detectors, made of novel materials and small crystals, and promotion of computer processing capacity for data analysis have been dramatically improved (4-6).

Time-of-flight (TOF) reconstruction, which is based on the determination of flight time lag between two annihilation gamma rays, is used for improving the spatial resolution and signal-to-noise ratio of images (4). Furthermore, incorporation of corrections methods with point spread function (PSF) into an iterative reconstruction algorithm can improve the contrast and spatial resolution of images, resulting in higher visibility of the target uptake (5-9). However, it has been pointed out that, with the use of the PSF method, the quantitative measurement of the standardized uptake value (SUV) should be interpreted carefully because of a potential overestimation (10-12). In order to make further progress, new techniques should be used positively. Previous phantom examinations have been conducted mainly at the center of the field of view (FOV). However, few researchers have clinically evaluated SUV as a function of the spatial position of a lesion within FOV.

In the present study, by utilizing TOF reconstruction and/or PSF correction, we examined the effect of the spatial position of lesions from the FOV center in the radial direction. We also evaluated the accuracy of SUV determination in phantom images and clinical cases.

## Materials and Methods

A Discovery PET/CT 710 system (GE Healthcare, Milwaukee, Wisconsin, USA) with 16-slice helical CT was utilized in the present study. The PET detector, equipped with a lutetium-based scintillator (4.2×6.3×25 mm^3^), could produce 47 slices per bed position with 15.7cm FOV in Z axis. The slice thickness was 3.27 mm, the diameter of the actual FOV for a slice was 700 mm, and the spatial resolution at 10 mm distance from the FOV center was 4.7 mm (13).

The used phantom was a JSP cylinder type Z4492-1994 (Kyoto Kagaku Corp., Kyoto, Japan), with a total interior volume of 6.35 l. We used CRC-25 PET Dose Calibrator (Capintec Inc. Ramsey, New Jersey, USA) to measure the amount of radioactivity. The image matrix size was 256×256. Image reconstruction was performed, using the ordered subsets expectation-maximization (OS-EM) algorithm with three iterations and 18 subsets. A Gaussian filter of 3.0 mm full width at half maximum (FWHM) was utilized as a post-smoothing filter. CT scans were acquired at 120 kV with automatic tube current modulation, 0.5 s tube rotation and 2.5 mm slice thickness. The Advantage Workstation version 4.6 (GE Healthcare, Milwaukee, Wisconsin, USA) was used for data analysis.

**Figure 1:**
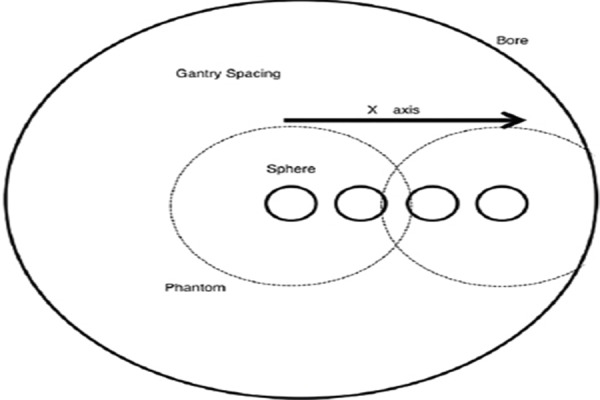
An overview of cylinder phantom study with regard to the location of the sphere, shifted from the field of view (FOV) center

In order to maintain the target-to-background ratio of radioactivity at 4:1, we prepared a 5.3 kBq/ml concentration of ^18^F-FDG for the background in the cylinder phantom and ^18^F-FDG concentration of 21.2 kBq/ml for the sphere (30 mm in diameter), located in the center of the cylinder phantom. The acquisition time was set at 180 sec, which was similar for all clinical examinations. SUV was determined by moving the phantom in a horizontal direction (X direction) from the FOV center (0 mm) at 50, 100,150 and 200 mm positions (Figure 1).

In total, 41 patients (23 men, 18 women, mean age: 68±11.2 years) were selected among patients with tumors, who had undergone PET-CT examinations between April 2014 and December 2014. The other requirements for inclusion was that the location of the tumors were minimally affected by breathing and the tumors were spatially separated from other uptake areas. For instance, tumors in the neck region and axillary or inguinal lymph nodes were evaluated in our study (Table 1).

**Table 1:** Initial diagnosis for 41 clinical cases

Comparison site	Number of cases	Mean Distance from FOV center (mm)
Cervical lymph node	20	53.5
Infraclavicular lymph node	9	44.2
Axillary lymph node	7	50.1
Inguinal lymph node	5	75.8
Total	41	53.7±25.2

**Figure 2:**
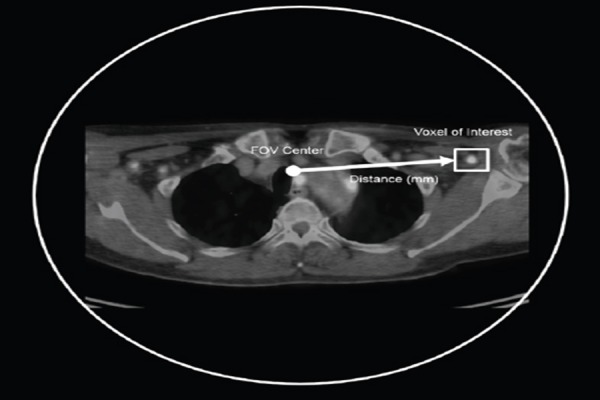
Illustration of the volume of interest (VOI) and the tumor distance from the field of view (FOV) center in a clinical case

All patients fasted for six hours prior to the examinations. The injected dose of ^18^F-FDG was 4.21±1.6 MBq/kg, and PET/CT scans were acquired 65 min following the injection. The size of tumors and their distance from the FOV center were measured, based on the clinical images (Figure 2).

We defined a volume of interest (VOI) for ^18^F-FDG accumulation of radioactivity by using TOF reconstruction and PSF correction methods. The VOI setting of SUV_max_ was defined as sufficient accumulation area. For the VOI setting of SUV_mean_, a 42% threshold was designated (14, 15). Afterwards, we determined the changes in SUV_max_ and SUV_mean_ at each VOI. Furthermore, variations in SUV due to the additional use of PSF correction were determined as follows:


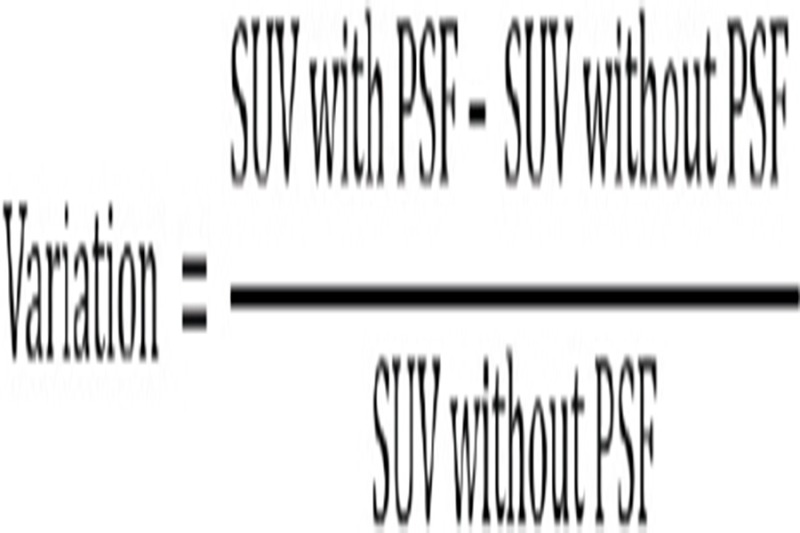


Changes in SUV in clinical cases were investigated, based on the distance of the tumor from the FOV center and tumor size. This study was approved by the ethics committee of the facility. In all cases, informed consent forms, which were prepared in accordance with ethical protocols, were obtained from the patients.

## Results

**Figure 3:**
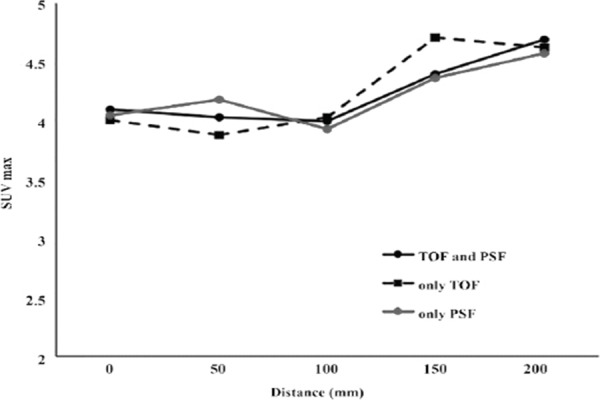
Effect of the location of simulated lesions on SUV_max_ by utilizing time of flight (TOF) reconstruction and/or point spread function (PSF) correction

Figure 3 presents SUV values for the 30 mm diameter sphere, as the cylinder phantom was moved outwards from the FOV center to a 200 mm distance in 50 mm increments. The SUV_max_ remained close to the theoretical value of 4.0 up to the 100 mm distance and then significantly exceeded the theoretical value beyond 100 mm distance.

**Figure 4:**
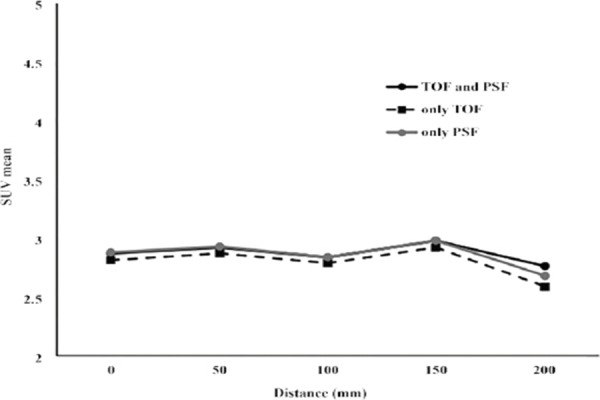
Effect of the location of simulated lesion on SUV_mean_ by utilizing time of flight (TOF) reconstruction and/or point spread function (PSF) correction

Similarly, SUV_mean_ remained close to the theoretical value up to 100 mm distance, while a slight increase was reported at 150 mm distance and a decline at 200 mm distance (Figure 4). It should be noted that the quantitative values of SUV_max_ and SUV_mean_ were not accurate beyond 100 mm displacement, even by applying TOF reconstruction and/or PSF correction methods.

**Figure 5:**
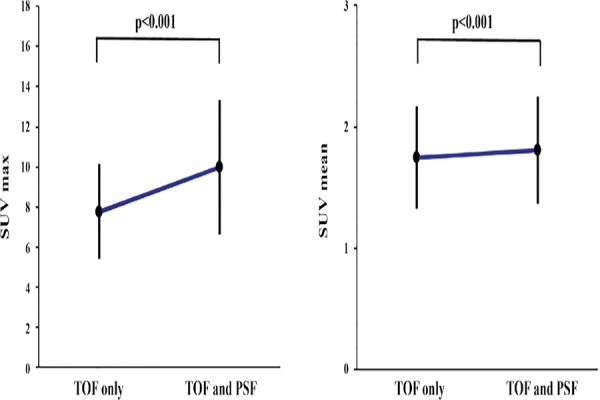
Effect of the concomitant use of time of flight (TOF) and point spread function (PSF) on SUV_max_ and SUV_mean_ in 41 clinical cases

**Figure 6:**
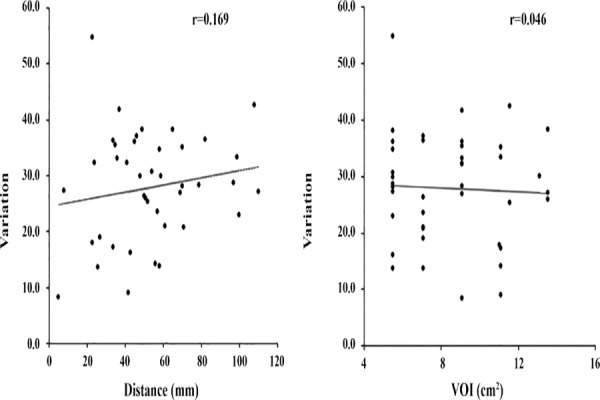
Effect of lesion location and volume of interest (VOI) size on SUV_max_ by the concomitant use of time of flight (TOF) and point spread function (PSF) in 41 clinical cases

**Figure 7:**
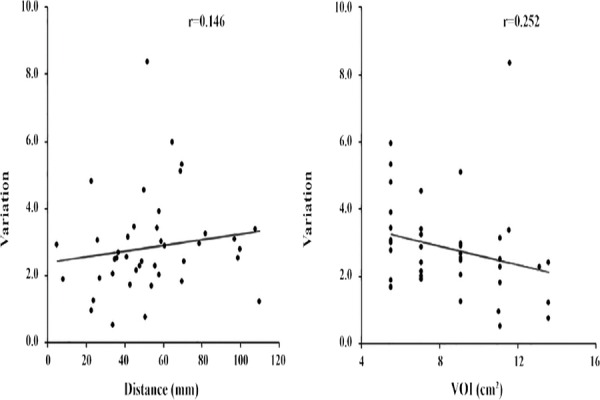
Effect of lesion location and volume of interest (VOI) size on SUV_mean_ by the concomitant use of time of flight (TOF) and point spread function (PSF) in 41 clinical cases

In clinical cases, changes in SUV_max_ and SUV_mean_ by the sole use of TOF reconstruction and concomitant use of both TOF reconstruction and PSF correction are demonstrated in Figure 5. Based on the findings, use of PSF reconstruction in addition to TOF significantly increased both SUV_max_ and SUV_mean_. The variations in SUV_max_ and SUV_mean_ in clinical cases as a function of distance from the FOV center are presented in Figures 6 and 7.

The average VOI size was calculated at 8.5 cm^3^. The addition of PSF correction increased SUV_max_ and SUV_mean_ by 27.9% and 2.8%, respectively. The distance from the FOV center did not affect SUV_max_ or SUV_mean_. On the other hand, SUV_mean_ tended to decrease slightly as the VOI increased. However, in both clinical cases, the distance between the tumor and FOV center was usually less than 100 mm. It should be noted that no significant change occurred by tumor displacement from the FOV center.

## Discussion

In this study, by applying TOF reconstruction and/or PSF correction methods, we examined whether the distance of lesions from the FOV center may affect SUV, based on the analysis of clinical cases and phantom study. Previous studies by the quantitative analysis of PSF correction have discouraged the use of this method due to SUV overestimation (11, 12). Therefore, it has been suggested that this method be applied with caution or discarded completely.

On the other hand, multiple studies have revealed the clinical advantage of PSF correction, considering its improved signal-to-noise ratio (7-10). In fact, by optimizing reconstruction parameters in combination with TOF reconstruction, the diagnostic accuracy may be improved, which can contribute to early cancer detection.

Previous studies on PSF correction have used a NEMA IEC body phantom with six simulated ^18^F-FDG accumulations (12, 16, 17). In these studies, the center of the

NEMA IEC body phantom was placed at the FOV center, following the ^18^F-FDG PET-CT imaging protocol (18-20). Therefore, the center of the sphere, i.e., six simulated lesions as the targets, was positioned at a 60 mm distance from the FOV center. In addition, the anterior surface of the phantom provided a thorax-like curve, simulating the human body structure.

The mentioned sphere location was deemed to produce different background factors at different locations. Therefore, in the present study, we placed the sphere in the middle of the cylinder phantom so that the background factor would remain constant in all directions; the size of the sphere was 30 mm. This condition was determined, based on our initial studies on phantoms (18, 20).

The SUV_max_ of the sphere was approximately in agreement with the theoretical value of 4.0 at a 100 mm distance from the FOV center. We believe that it is reasonable to select a sphere with a 30 mm diameter for cylinder phantom studies. Furthermore, the concomitant use of PSF correction did not cause a change in SUV_max_ within a 100 mm distance from the FOV center, whereas SUV_max_ was overestimated at greater distances. The SUV_mean_ did not change within a 100 mm distance from the FOV center, whereas an underestimation was reported at distances beyond 100 mm.

Use of TOF reconstruction and PSF correction methods facilitated the quantitative evaluation of SUV for ^18^F-FDG accumulation in a 100 mm distance from the FOV center. An increase was reported in SUV due to the application of PSF correction in all clinical cases; however, the tumor location from the FOV center did not affect SUV. Based on our cylinder phantom study, we had anticipated the effect of PSF correction on tumors distant from the FOV center in clinical cases. Therefore, we compared ^18^F-FDG accumulation in neck regions and axillary and inguinal lymph nodes, which are far from the trunk center.

For most lesions located within a 100 mm distance from the FOV center, PSF correction did not significantly affect SUV_max_. However, SUV_mean_ was shown to be affected to some extent, depending on the tumor size and its location. As determined in this study, although SUV_max_ is often used for evaluating tumors in actual clinical cases, careful examinations are required while calculating SUV_mean_ as a reference. Based on the present study, tumor locations farther than a 150 mm distance from the FOV center are uncommon.

This study had certain limitations. In some cases, tumors are located at a 100 mm distance (or farther) from the FOV center. Therefore, further studies are required for detailed evaluation of such tumors, which are distant from the FOV center. Furthermore, detailed examination of spatial resolution with regard to tumor distance from the FOV center is required.

## Conclusion

Based on our findings, when a tumor was located at a 100 mm distance (or farther) from the FOV center, the reliability of the quantitative value of SUV, obtained by TOF reconstruction and/or PSF correction methods, decreased. In clinical settings, with the use of both TOF and PSF reconstruction methods, SUV_max_ remained stable within a constantly rising range, if the tumor of lymph nodes was located within a 100 mm radius from the FOV center. Overall, evaluation by both TOF and PSF can be helpful if these findings are taken into account. Improvements in image contrast and detectability may contribute to enhancements in the detection of small lesions.

## Conflicts of interest

The authors declare no conflicts of interest.
